# Hyperspectral Imaging Combined with Multi-Scale Feature Fusion and Improved Faster R-CNN for Automated Detection of Surface Defects on Chicken Carcasses

**DOI:** 10.3390/foods15162775

**Published:** 2026-08-07

**Authors:** Huihui Wang, Siyuan He, Kangyi Ding, Yuanshan Zhao, Wenkai Wang, Yang Wang, Xu Zhang

**Affiliations:** 1 School of Mechanical Engineering & Automation, Dalian Polytechnic University, Dalian 116034, China; whh419@126.com (H.W.); 13470463101@163.com (S.H.); 18623761928@163.com (K.D.); zhaoyuanshan2024@163.com (Y.Z.); 18317736585@163.com (W.W.); 18340826853@163.com (Y.W.); 2 National Engineering Research Center of Seafood, Dalian Polytechnic University, Dalian 116034, China; 3 State Key Laboratory of Marine Food Processing and Safety Control, Dalian Polytechnic University, Dalian 116034, China

**Keywords:** defect detection, hyperspectral imaging, deep learning, chicken

## Abstract

Chicken meat holds a dominant position in the meat consumption market, and broilers are important primary processed products. However, during the slaughtering and processing of broilers, steps such as hanging, bleeding, scalding, defeathering, neck slitting, and evisceration can easily cause skin break and skin scratch on chicken carcasses due to manual operational errors. Additionally, chicken carcasses may suffer broken wing bones from intense collisions during transportation. These issues negatively impact the quality and market acceptance of broilers in subsequent sales. This study aims to develop a stable and efficient method that leverages the unique technical advantages of hyperspectral imaging information fusion and rich dimensionality. By capturing, processing, and fusing multi-scale information, the method achieves automatic detection of defects such as skin break, skin scratch, and exposed broken bones during chicken carcass processing. The improved defect detection model for chicken carcasses shows significantly enhanced performance, with average precision (AP) exceeding 92% and recall rates above 91% for all defects. The overall mean average precision (mAP) increased from 80.2% to 93.6%. This method not only provides a reliable technical approach for detecting defects in chicken carcasses but also offers a referable solution for the visual inspection of other livestock and poultry products.

## 1. Introduction

As the second largest consumed meat globally [1,2], chicken occupies a major position in the meat consumption market. Chickens are important primary processed products, and their integrity is a crucial factor influencing the evaluation criteria of chicken meat. However, during the processing steps of broiler slaughter, such as hanging, bleeding, scalding, plucking, neck skin incision, and evisceration [3], the use of sharp tools often leads to the formation of thin, elongated scars on the surface of the chicken carcass. In addition, improper handling during scalding or plucking procedures may result in large-scale skin tearing or even detachment of the chicken carcass. Meanwhile, during handling or processing, the chicken carcass may suffer from severe impacts, leading to wing fractures, the formation of sharp bone spurs that pierce the skin, and the exposure of broken bones. Skin break and skin scratch may affect the storage quality of the chicken carcass, while exposed broken bones could pose a food safety risk. These defects not only diminish the market appeal of the chicken but also reduce consumers’ willingness to purchase [4]. More importantly, the detection of chicken carcass defects still largely relies on manual inspection. In practical poultry processing, defect inspection requires not only determining whether a carcass is defective, but also accurately identifying the defect category and locating the defective region. However, defects such as skin scratches, skin breaks, and exposed bones are difficult to observe and distinguish due to their blurred boundaries and low contrast with surrounding tissues, resulting in high false detection and missed detection rates. Furthermore, as modern slaughter lines process thousands of chicken carcasses per hour, inspectors are prone to fatigue and oversight, leading to inconsistent inspection results and reduced production efficiency. Therefore, developing an accurate, non-destructive, and objective method for detecting and localizing chicken carcass defects has become a key technological requirement for improving poultry meat processing quality.

In recent years, machine vision, with its advantages of fast and non-destructive detection, has been applied to various broiler processing stages, including automatic identification of dead chickens [5] and broiler weight prediction [6]. However, the chicken tissue exposed by skin scratches and skin breaks is very similar in texture and color to the surrounding healthy chicken skin, making it difficult for visible-light-based machine vision systems to effectively identify such defects. Additionally, traditional machine vision systems perform poorly in detecting exposed broken bones, as these quickly turn brown after exposure. The RGB-based analysis methods often confuse the brown color with residual blood on the carcass surface, leading to high recognition and false detection rates. Hyperspectral imaging (HSI) technology captures both 1D spectral data and 2D spatial images, enabling material identification and quantitative analysis of physicochemical properties, and providing detailed information on target categories and spatial distribution [7]. By integrating spectral information with physicochemical and spatial features, this technology significantly improves the accuracy and reliability of object detection in complex scenarios [8]. HSI has been successfully applied in studies such as red meat adulteration detection [9], surface foreign object detection in livestock [10], and distinguishing fresh from frozen beef [11], effectively overcoming the challenges faced by traditional machine vision, including difficulty in detecting similar traits and structures and high false detection rates in complex environments. These studies demonstrate the potential of HSI in meat quality detection, offering new solutions to the challenges faced by traditional machine vision.

Hyperspectral images are high-dimensional, information-dense data, making the effective extraction of relevant information and the construction of suitable analytical models crucial for accurate detection of chicken carcass defects. In chicken carcass defect detection, it is essential to remove redundant information from the vast amount of hyperspectral image data while also designing an appropriate model for the final detection. To improve the efficiency and speed of HSI data processing, deep learning, particularly Convolutional Neural Networks (CNNs), has become a commonly used method. CNNs offer unique advantages in handling high-dimensional complex data and have gradually been integrated with hyperspectral imaging technology [12]. Recent machine learning approaches, including neural networks, Gaussian process regression, graph-based models, and ensemble learning, have shown strong potential for nonlinear modeling in agricultural and food science applications. However, chicken carcass defect inspection requires not only category discrimination but also precise spatial localization. Therefore, compared with general classification or regression models, an object detection framework is more suitable for this task. Faster R-CNN was selected because of its strong localization accuracy and stable performance for small, low-contrast targets, which matches the detection requirements of skin scratches, skin breaks, and exposed broken bones. Specifically, in the chicken carcass processing field, CNN has shown promising application prospects, such as predicting the moisture, protein, and ash content in chicken meat, detecting foreign objects in chicken meat [13], and identifying the freshness of chicken meat [14]. However, existing research mostly focuses on composition detection or quality grading. There is still a lack of effective automated detection solutions for appearance defects, particularly small-sized, low-contrast skin scratches and skin breaks, which directly impact product value and safety. This research gap significantly limits the poultry processing industry’s ability to precisely control product quality. Therefore, enhancing the small target recognition capability within the CNN framework and optimizing its performance in detecting small defects on chicken carcasses is essential.

**Figure 1:**
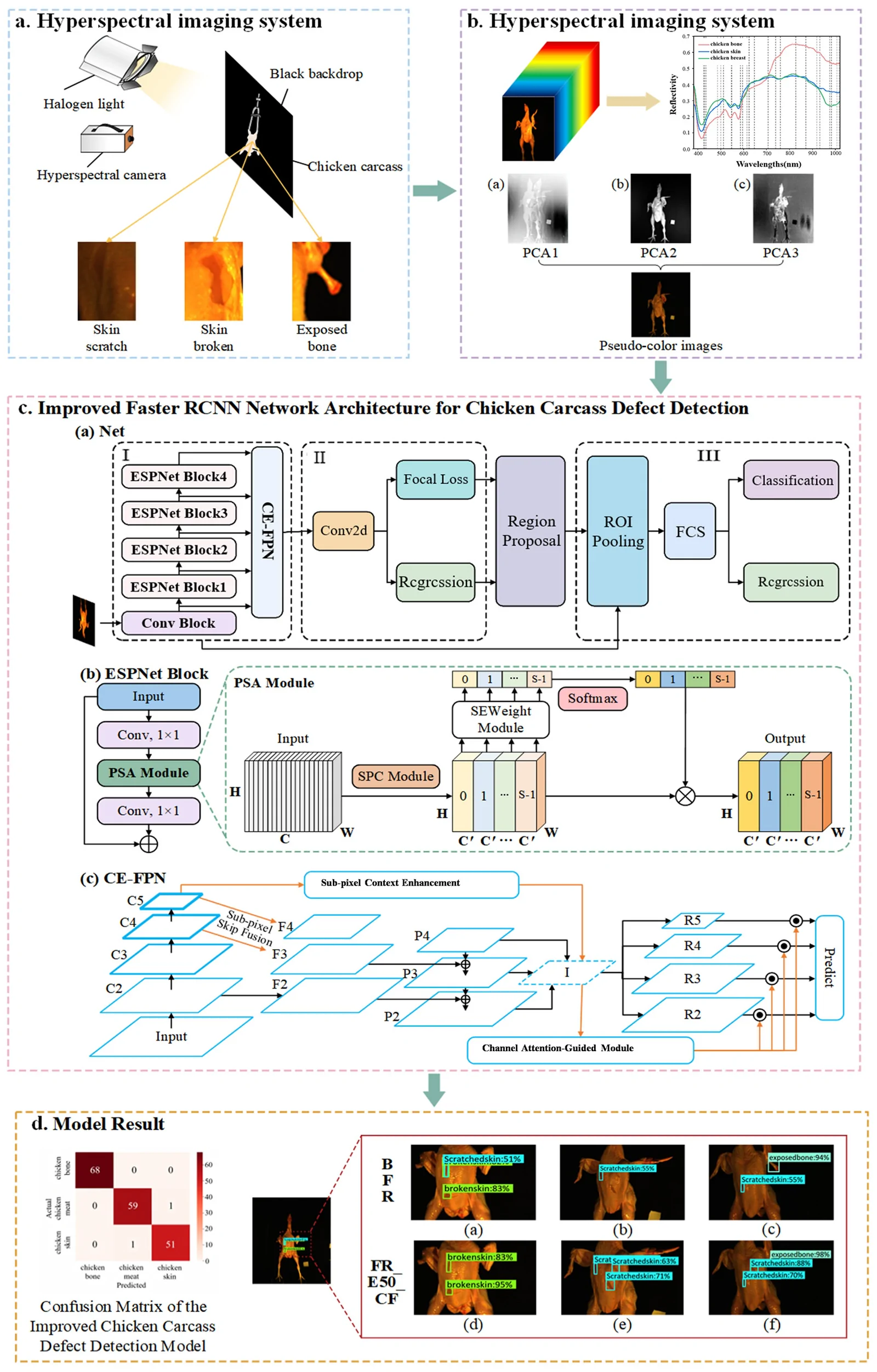
Experimental Workflow: (**a**) Hyperspectral Imaging System, (**b**) Hyperspectral Data Dimensionality Reduction, (**c**) Improved Faster R-CNN Chicken Carcass Defect Detection Model, (**d**) Model Detection Results.

This study aims to deeply integrate deep learning and HSI to develop an efficient and robust method for chicken carcass defect detection, precisely identifying and locating three key defects: skin breaks, skin scratches, and exposed broken bones. To achieve this, the study systematically carried out the following tasks: (1) It constructed a professional and comprehensive datasets of skin scratches, skin breaks, and exposed broken bones to lay the foundation for model training and validation. (2) It proposed a data processing pipeline combining the CARS algorithm and principal component analysis (PCA), aimed at effectively extracting feature bands from high-dimensional spectral data and generating high-quality pseudo-color images to optimize the input information for subsequent models. (3) To address the challenges of small defect detection, it improved the Faster R-CNN framework by introducing the EPSANet50 backbone network to enhance feature representation. Additionally, it integrated a Channel-Enhanced Feature Pyramid Network (CE-FPN) to strengthen multi-scale feature fusion, particularly for small-sized defects. This led to the development of a high-performance chicken carcass surface defect detection model, achieving high-precision identification and detection of skin scratches, skin breaks, and exposed broken bones. The specific research content is shown in Figure 1.

## 2. Materials and Methods

### 2.1. Sample Preparation

This study focused on Phoenix chicken carcasses. The samples were sourced from different farms; 10 batches were randomly selected from a 20-day production cycle at the slaughterhouse, with approximately 50 carcasses randomly selected from each batch. Although these chickens came from different farms, they belonged to the same breed and were processed under the same industrial slaughter conditions. All chicken carcasses underwent the same slaughtering and hyperspectral imaging procedures. After excluding samples with poor image quality or incomplete information, a total of 466 chicken carcasses were ultimately retained for subsequent analysis.

All samples were manually pre-screened and confirmed to have defects such as skin scratches, skin breaks, or exposed broken bones. To build a comprehensive sample database, images were captured for both the front and back of each sample, resulting in a total of 932 hyperspectral images. The final dataset contains 373 cases of skin scratches, 171 cases of skin breaks, and 442 cases of exposed broken bones. The hyperspectral images were divided into training, validation, and testing sets at a ratio of 7:2:1. For samples with composite defects, each defect instance was annotated separately according to its defect category. For example, when skin scratches and exposed bone appeared on the same carcass image, independent bounding boxes were drawn for each defect region and assigned the corresponding labels. Therefore, one image could contain multiple bounding boxes and multiple defect categories. During model training and evaluation, each annotated defect instance was treated as an independent target. Each image in the dataset contains at least one annotated defect, but most of the image consists of intact chicken skin and normal tissue. During Faster R-CNN training, these normal regions are treated as background and classified as negative samples, enabling the model to learn to distinguish between defect regions and normal tissue. Therefore, this dataset is suitable for supervised defect detection and provides ample background information for model optimization. During rearing, transport, and slaughter, most skin injuries and scratches occur primarily on the chest of chicken carcasses. Therefore, this area was selected as the representative muscle tissue for subsequent spectral analysis.

### 2.2. Hyperspectral Imaging System

The experimental equipment used in this study is shown in Figure 1a. The system consists of a visible–near-infrared hyperspectral camera (Image-V10E-HR, Sichuan Shuangli Spectral Imaging Technology Co., Ltd., Chengdu, China) and a display. The hyperspectral camera consists of a built-in microcomputer, an acousto-optic tunable filter (AOTF), a charge-coupled device (CCD) detector, and a data acquisition and control module. The hyperspectral system is set up as follows: the resolution of single-band spectral images is set to 1344 × 1024 pixels^2^, the wavelength range is 380.2~1020.2 nm, the spectral resolution is set to 1.8 nm, and the exposure time is 15 ms.

### 2.3. Hyperspectral Data Preprocessing

Due to factors such as current stability, light source placement, and light intensity, the hyperspectral imaging system may introduce dark current and noise into the images. To minimize the above interference, hyperspectral images typically require black-and-white calibration. The black calibration image is obtained by turning off the light and covering the camera lens. The white calibration image is acquired by capturing an image of the calibration white cloth. The calibration is performed using the following formula:

(1) $$I_{ref}=\frac{I-B}{W-B}$$

where *I*ref is the calibrated data, *I* is the original image, *B* is the black calibration image, and *W* is the white calibration image.

### 2.4. Scanning Electron Microscope (SEM) Characterization

The microstructure of chicken breast, chicken skin, and chicken bones was observed using a Scanning Electron Microscope (SEM) (JEOL Ltd., Tokyo, Japan). The chicken breast and chicken skin were cut into rectangular cubes measuring 5 × 5 × 3 mm^3^, and the chicken wings, after deboning, were also cut into the same specifications. All samples were fixed with glutaraldehyde solution, followed by sequential dehydration using ethanol solutions of varying concentrations. After dehydration, the samples were freeze-dried and metal-coated using ion sputtering. The microstructure was characterized and spectra were acquired using a JSM-7800F Scanning Electron Microscope (JEOL Ltd., Tokyo, Japan).

### 2.5. Data Dimensionality Reduction

Hyperspectral images contain a large amount of redundant information, which not only increases computational complexity but also poses challenges for small-target detection [15]. The skin scratches, skin breaks, and exposed bone defects on the chicken carcass, which are the focus of this study, exhibit small-target characteristics. Their spectral features are easily affected by redundant bands and are difficult to capture during imaging due to changes in lighting or viewing angles. To remove redundancy and efficiently extract key spectral features, this study employs the Competitive Adaptive Reweighted Sampling (CARS) algorithm for spectral data dimensionality reduction. However, defect detection requires not only identifying the type but also precise spatial localization. The defect features in the original single-band images are weak, making them difficult to directly use for localization. Although the feature bands selected by CARS contain discriminative information, they have not been fully transformed into spatially distinguishable features. Therefore, this study further applies Principal Component Analysis (PCA) to the feature band images obtained through CARS, in order to enhance the morphological, positional, and boundary features of the defects in the spatial dimension, providing a basis for precise defect localization. Based on this, the top three principal component images with the highest contribution rates are selected, and pseudo-color images are generated through RGB fusion to further enhance the spatial distinguishability of the defects. All pseudo-color images are used to build the chicken carcass defect model dataset.

### 2.6. Experimental Environment

Regarding the data in this paper, hyperspectral images were processed using ENVI 5.6 software, and the proposed deep learning model was developed in Python 3.8. The Faster R-CNN-based detection framework was implemented using the PyTorch 1.13 deep learning framework. The model was developed and trained using PyCharm 2021 as the integrated development environment. All experiments were conducted on a computer running Windows 10, equipped with an Intel Core™ i7-11800H @ 2.30 GHz processor and an NVIDIA GeForce RTX 4070 GPU with 12 GB of video memory. A GPU-accelerated environment was configured to improve the efficiency of model training and inference.

### 2.7. Enhanced Faster R-CNN Model for Chicken Carcass Defect Recognition Aimed at Small Target Detection

**Figure 2:**
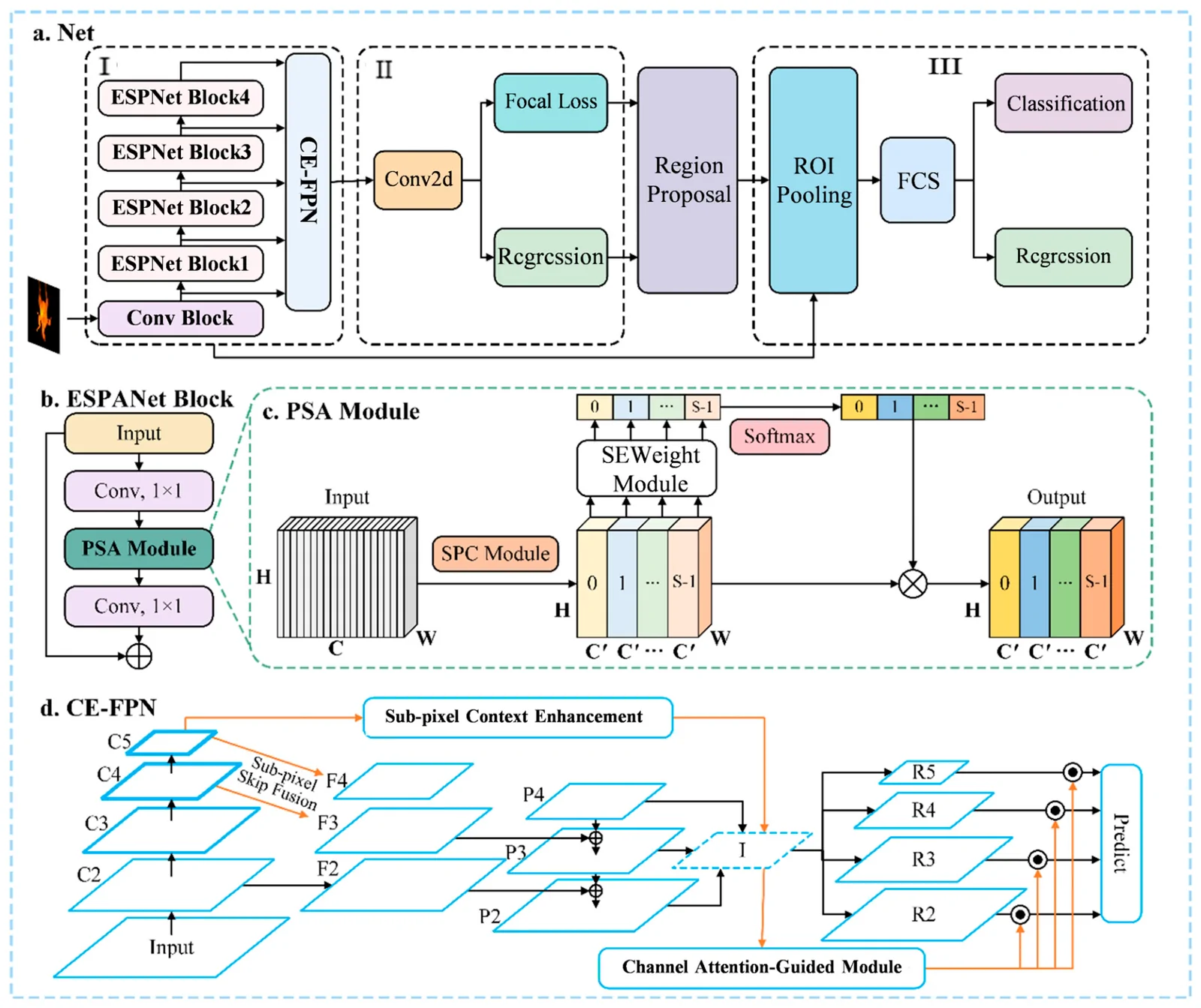
(**a**) Structure of the improved chicken carcass defect detection model based on Faster R-CNN: (I) Feature Extraction Network, (II) Region Proposal Network, (III) Prediction Network; (**b**) diagram of the EPSANet Network; (**c**) structure of the Pyramid Squeeze Attention (PSA) mechanism; (**d**) Diagram of the Channel Enhanced Feature Pyramid Network (CE-FPN).

Faster R-CNN, as a classic two-stage object detection algorithm, has demonstrated strong potential in food quality monitoring and has been successfully applied to the identification of defects such as foreign objects in fruits and vegetables [16], surface lesions in agricultural products [17], and meat freshness [18]. In this study, surface defects on chicken carcasses (such as skin scratches, skin breaks, and exposed broken bones) appear as typical small targets within the overall image. The detection of small targets largely depends on the ability to capture detailed features [19]. The skin scratches and skin break areas are extremely similar in color and texture to the surrounding normal skin, making it difficult to effectively distinguish their subtle differences during feature extraction. Therefore, a specialized network structure that focuses on fine details is required for accurate differentiation. In addition, the exposed broken bone defect varies significantly in morphology and size, which further increases the difficulty for the network to extract stable and discriminative features from its variable contours. To address the above challenges, this study proposes an improved Faster R-CNN model for chicken carcass defect detection. As shown in Figure 2, the model consists of three parts: the feature extraction network, the region proposal network, and the prediction network. The improved feature extraction network is shown in Figure 2a. In this study, EPSANet50 is used as the backbone network, and a Channel-Enhanced Feature Pyramid Network (CE-FPN) is introduced. Both work together to adaptively extract and enhance multi-scale features of chicken carcass defects, particularly the fine features of small targets. The Region Proposal Network (RPN, Figure 2b) utilizes the multi-level feature maps output by the feature extraction network. Through the anchor mechanism, it generates a series of candidate regions where defects are likely to exist, thus performing initial screening and coarse localization of the defect areas. The prediction network (Figure 2c), as the final decision module, combines the candidate boxes provided by the Region Proposal Network with the deep features extracted by the feature extraction network. It performs joint analysis to output both the classification results for the three defects—skin scratches, skin breaks, and exposed broken bones—and the precise boundary box localization.

#### 2.7.1. EPSANet50 Feature Extraction Network

In the original Faster R-CNN framework, the feature extraction network is responsible for extracting multi-level features of the image through a deep convolutional neural network. As the network depth increases, deeper networks can extract increasingly complex features, but this may also lead to the loss of some shallow detail information. For defects such as skin scratches and skin breaks on the chicken carcass, their appearance changes are subtle and the contrast is low, making it easy for deep networks to overlook these low-variance, small features. To address the above issue, this study reconstructs the chicken carcass defect feature extraction part using EPSANet50. The improved network architecture is shown in Figure 2. EPSANet is a lightweight network based on the ResNet architecture [20], as shown in Figure 2b. This network replaces the 3 × 3 convolution in the ResNet bottleneck block with the PSA algorithm (as shown in Figure 2b), forming an efficient pyramid squeeze attention mechanism. The PSA algorithm, when learning features, can focus on the tiny tears and damage in skin breaks, improving the detection accuracy of small-area defects. It also enhances the perception of scratch direction, capturing texture information related to the scratches. In addition, it can increase the model’s sensitivity to the local exposure features of broken bones, reducing the interference from the background in exposed broken bone detection. As shown in Figure 2c, the PSA algorithm performs channel segmentation and feature extraction through the SPC structure. Then, it uses the SEWeight algorithm to extract channel attention vectors, recalibrates the attention weights using the Softmax function, and finally multiplies the recalibrated weights with the feature map to achieve adaptive feature enhancement.

#### 2.7.2. Channel-Enhanced Feature Pyramid Network (CE-FPN)

In deep convolutional networks, a series of convolution operations generate feature maps at different scales. However, due to the large spacing between deep convolutions, the feature maps in the final layers are prone to losing detailed information about chicken carcass defects. The Feature Pyramid Network (FPN) enhances the representation of multi-scale features by constructing a top-down path and fusing it with lower-level features, which helps in extracting semantic information for small targets. However, traditional FPN networks use unidirectional feature fusion, typically implemented through simple pixel-wise addition. This can lead to aliasing effects during cross-scale fusion, causing feature confusion and increased false detection rates. To overcome the above limitations, this study introduces the Channel-Enhanced Feature Pyramid Network (CE-FPN) into the chicken carcass defect feature extraction network. As shown in Figure 2d, this module takes the feature maps (C2, C3, C4, C5) output at different stages of EPSANet50 as input. First, through the Subpixel Skip Fusion (SSF) module, C4 and C5 are fused to generate F4, C4 and C3 are fused to generate F3, and C2 is directly processed with a 1 × 1 convolution to obtain F2. To avoid aliasing interference, the path originally generating P5 from C5 is removed. Then, in the top-down path, P4 corresponds to F4, which is upsampled and sequentially fused with F3 and F2, gradually generating P3 and P2. Finally, the features extracted from the above sections are fused and mapped to the feature map layer I. To further suppress aliasing and enhance feature selectivity, this structure introduces an attention-guided mechanism (CAG) to improve the network’s ability to fuse multi-scale features related to chicken carcass defects [21]. After passing through the CAG module, feature map I is assigned different weights for each feature layer (R2, R3, R4, R5), ultimately constructing a weighted multi-scale feature pyramid. CE-FPN enhances the network’s ability to fuse features related to chicken carcass defects at different scales, enabling the model to fully utilize both high-level semantic information and low-level feature information of the defects.

### 2.8. Evaluation Metric 

To objectively and comprehensively evaluate the established model, Recall, Precision, Average Precision (AP), and mean Average Precision (mAP) were used as evaluation metrics. The calculation formulas are as follows:

(2) $$Precision=\frac{TP}{\left(TP+FP\right)}$$

(3) $$Recall=\frac{TP}{\left(TP+FN\right)}$$

(4) $$AP=\int_{0}^{1}Precision\;d\left(Recall\right)$$

(5) $$mAP=\frac{AP}{C}$$

where TP (true positive) is the number of chicken carcass defect samples correctly predicted by the model for category *i*. The more TPs, the more accurate the recognition of that category. TN (true negative) is the number of chicken carcass defect samples correctly predicted by the model as not belonging to category i. The more TNs, the stronger the ability to exclude negative samples. FP (false positive) is the number of chicken carcass defect samples incorrectly predicted by the model as belonging to category i, while these samples actually do not belong to that category. The more FPs, the higher the likelihood of misjudgment. FN (false negative) is the number of chicken carcass defect samples that the model fails to predict as belonging to category i, while these samples actually belong to that category. The more FNs, the more severe the missed detection. *C* is the number of defect categories in chicken carcasses.

## 3. Results and Discussion

### 3.1. Hyperspectral Data Analysis

**Figure 3:**
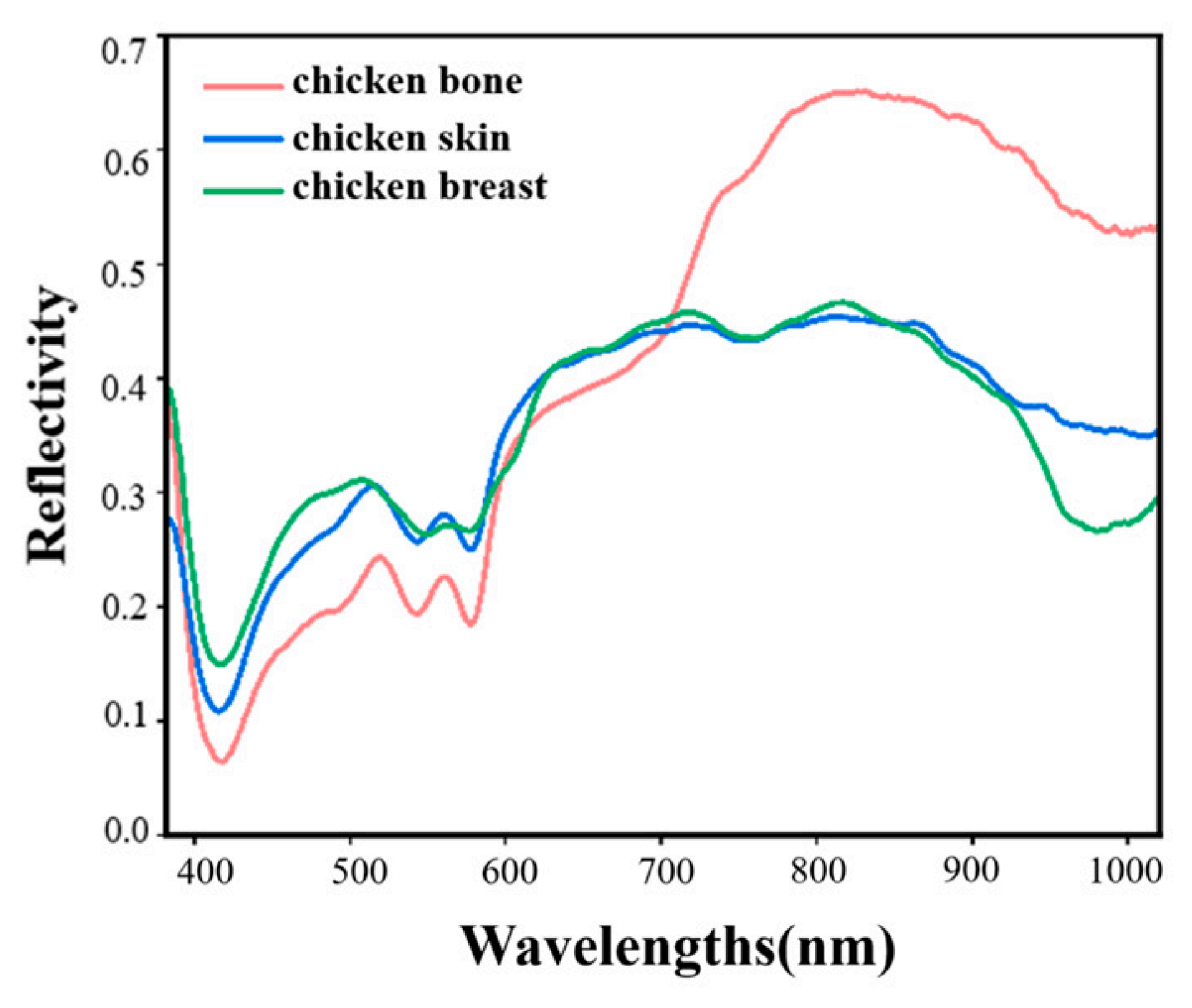
Spectral curves of chicken breast, chicken skin, and chicken bone.

Specific functional groups or chemical components exhibit selective absorption of light at specific wavelengths [22]. Hyperspectral reflectance curves capture the distinct spectral responses of different substances in the near-infrared region [23]. Therefore, effective dimensionality reduction and feature band selection are essential to minimize redundant information interference and improve recognition accuracy. Figure 3 presents the average spectral curves of regions of interest for chicken skin, chicken breast, and bone over the 360.2–1020.2 nm range. In the 400–580 nm region, the spectral curves of all three tissue types show similar trends: reflectance drops to a minimum around 410 nm, followed by two weak absorption peaks near 540 nm and 575 nm. The absorption peaks at these two points correspond to the characteristic absorption of deoxygenated hemoglobin and oxygenated hemoglobin [24]. The distinct absorption peaks in chicken breast reflect its high myoglobin content, whereas the weaker absorption in chicken skin is associated with residual hemoglobin in the dermal layer [25]. Beyond 580 nm, the spectral characteristics of skin, meat, and bone diverge significantly. The reflectance of skin and meat increases sharply to a peak in the 580–725 nm range, primarily due to stretching overtones of C-H and C-O bonds in proteins and fats. In addition, both skin and meat exhibit a characteristic absorption trough near 985 nm, attributed to collagen absorption. In contrast, the reflectance of bone increases more gradually and lacks the collagen-related absorption trough at 985 nm [26], consistent with its mineral-rich, collagen-poor composition. In the 900–1020 nm near-infrared region, the reflectance of skin and meat is significantly higher owing to the combined effects of fat and collagen, while the reflectance curve of bone remains relatively flat due to the absence of collagen. This divergence provides a reliable basis for bone identification within the near-infrared range. Furthermore, the spectral differences manifested by the absorption features in the 580–725 nm and 985 nm regions also help distinguish between chicken skin and meat, offering a spectroscopic foundation for subsequent classification modeling.

### 3.2. SEM Results Analysis

**Figure 4:**
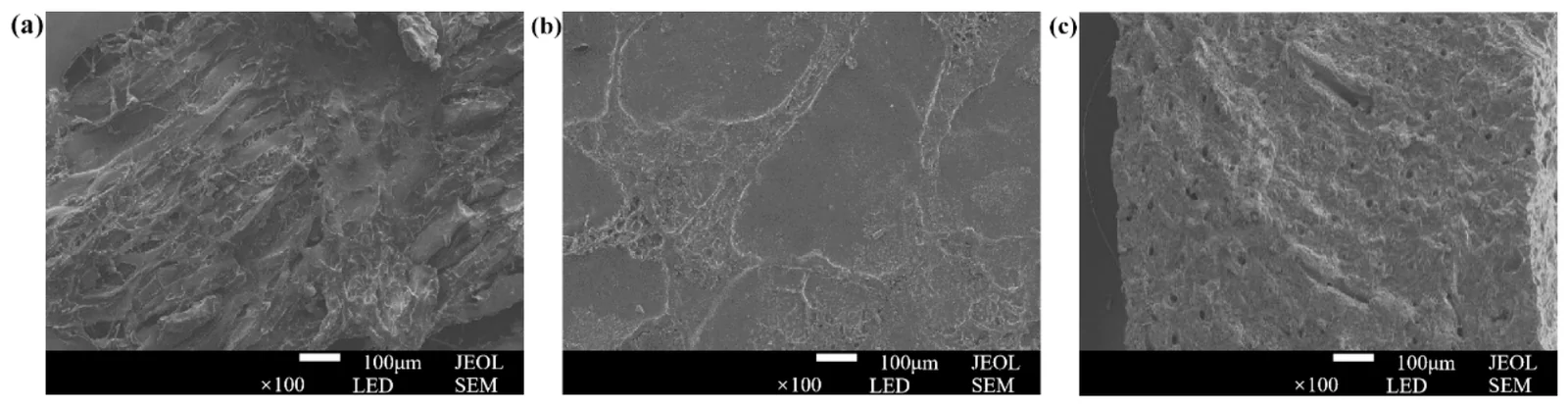
Scanning Electron Microscope Images: Chicken Breast (**a**), Chicken Skin (**b**), and Chicken Bone (**c**).

Figure 4 presents the scanning electron microscopy (SEM) images of chicken breast, skin, and bone. Figure 4a reveals the microstructure of chicken breast, characterized by tightly arranged muscle fibers aligned in a consistent direction with uniform inter-fiber spacing, forming a regular network. Some regions show a smoother morphology attributable to fat infiltration, which correlates with the distinct fat absorption peaks observed at 750 nm and 985 nm in the corresponding spectral curve (Figure 3). In contrast, the chicken skin tissue (Figure 4b) exhibits a generally flat and smooth surface with structural continuity and no obvious gaps. Comprising the epidermis and dermis, the skin has a higher collagen content and fewer muscle fibers in the dermal layer. This compositional difference accounts for the significantly weaker absorption intensity near 985 nm in the skin spectrum compared to that of meat, reflecting its specific collagen absorption behavior. The microstructure of chicken bone (Figure 4c) is predominantly composed of minerals, presenting a porous and rough surface. The trabecular structure is dense, compact, and interconnected, forming a network. Due to its mineral-dominated composition with negligible fat and collagen content, the corresponding spectral curve (Figure 3) shows no significant characteristic absorption responses in the 750–985 nm range. The above microstructural analysis demonstrates significant differences in tissue morphology and chemical composition among chicken breast, skin, and bone. These morphological and compositional variations are highly consistent with their respective hyperspectral features, thereby validating the effectiveness of spectral response for differentiation and providing a theoretical basis for the rapid, non-destructive identification of chicken carcass components using hyperspectral imaging technology.

### 3.3. Dimensionality Reduction Results Analysis

**Figure 5:**
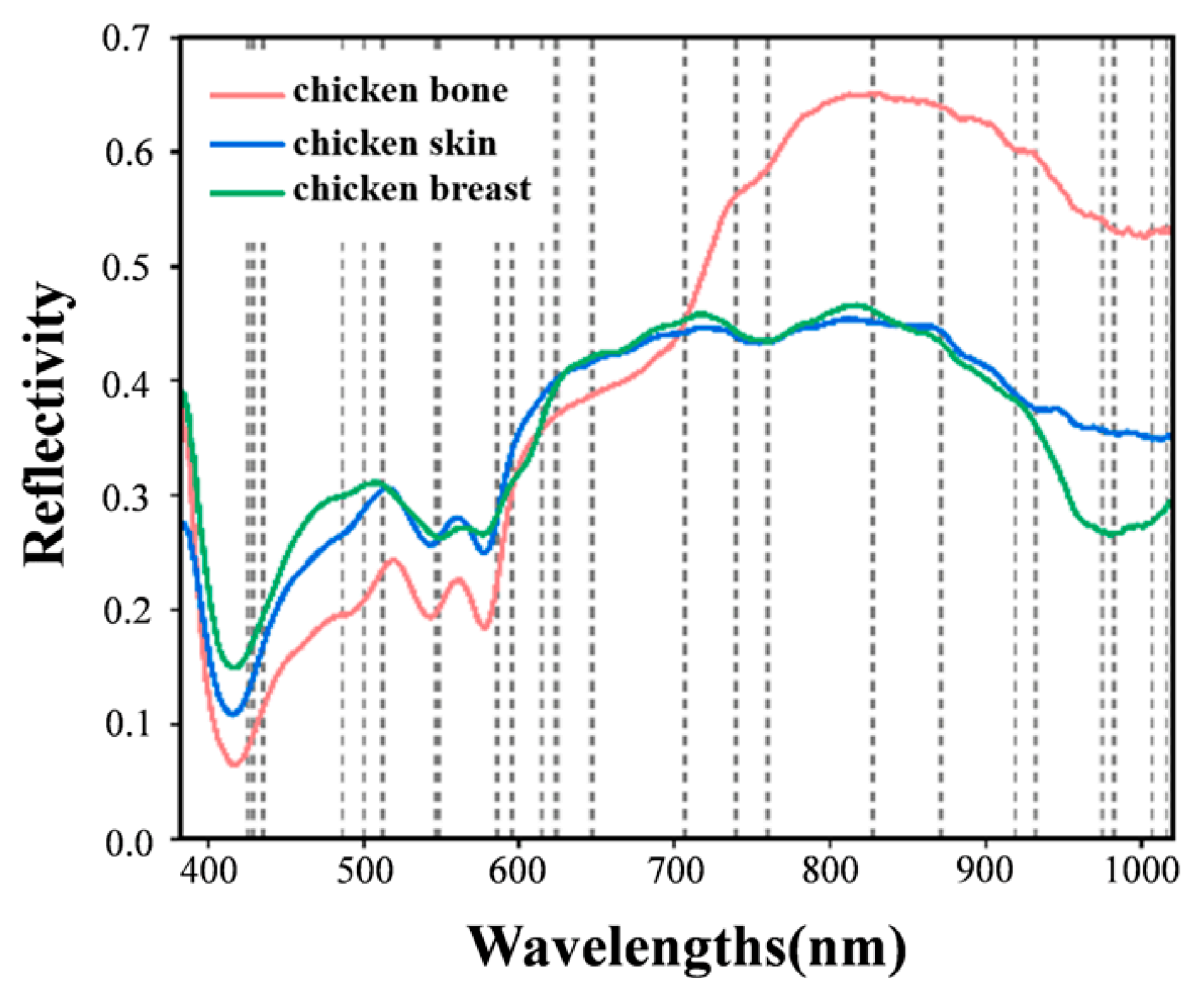
Feature wavelengths extracted by the Competitive Adaptive Reweighted Sampling (CARS) method.

In this study, the hyperspectral wavelength range is from 380.2 to 1020.2 nm, with a total of 360 bands. To reduce data redundancy and enhance feature representativeness, dimensionality reduction is required to represent the full spectral data using a few characteristic bands. Therefore, the CARS algorithm is used for spectral data dimensionality reduction in this study. The CARS algorithm ultimately selected 28 characteristic bands, as shown in Figure 5. These bands are primarily concentrated in the spectral ranges of 400–650 nm and 900–1020 nm. At the absorption peak around 550 nm, the CARS algorithm extracted two bands at 548.5 nm and 546.7 nm, which correspond to the absorption characteristics of hemoglobin and myoglobin, respectively [27]. These bands can be used to effectively differentiate between blood and bone tissues, providing a key spectral basis for subsequent classification. In addition, around 750 nm, the bands at 740.4 nm and 760.2 nm are attributed to protein absorption and C-H stretching vibration overtone of fat [28]. This result aligns with the distribution of fat observed between muscle fibers in the scanning electron microscope images (Figure 4a). In the 975.1–992.6 nm wavelength range, the spectral reflectance is more sensitive to moisture. Chicken skin shows a higher reflectance in this range, consistent with its relatively low water content. The scanning electron microscope results (Figure 4b) show that the dermis of chicken skin has a dense arrangement of collagen and a relatively smooth surface structure. This structure may, to some extent, limit its water retention capacity. No significant absorption response is observed in the 750–985 nm range for chicken bones, consistent with their composition, which contains almost no water. The porous, rough mineral structure observed in the scanning electron microscope image (Figure 4c) may correspond to its unique spectral characteristics. It can be seen that CARS effectively summarizes the spectral characteristics by selecting significant response points such as peaks, valleys, and shoulders in the spectral curve, using fewer bands to achieve a compact representation of the spectrum. This provides high-discriminatory input features for subsequent modeling.

**Figure 6:**
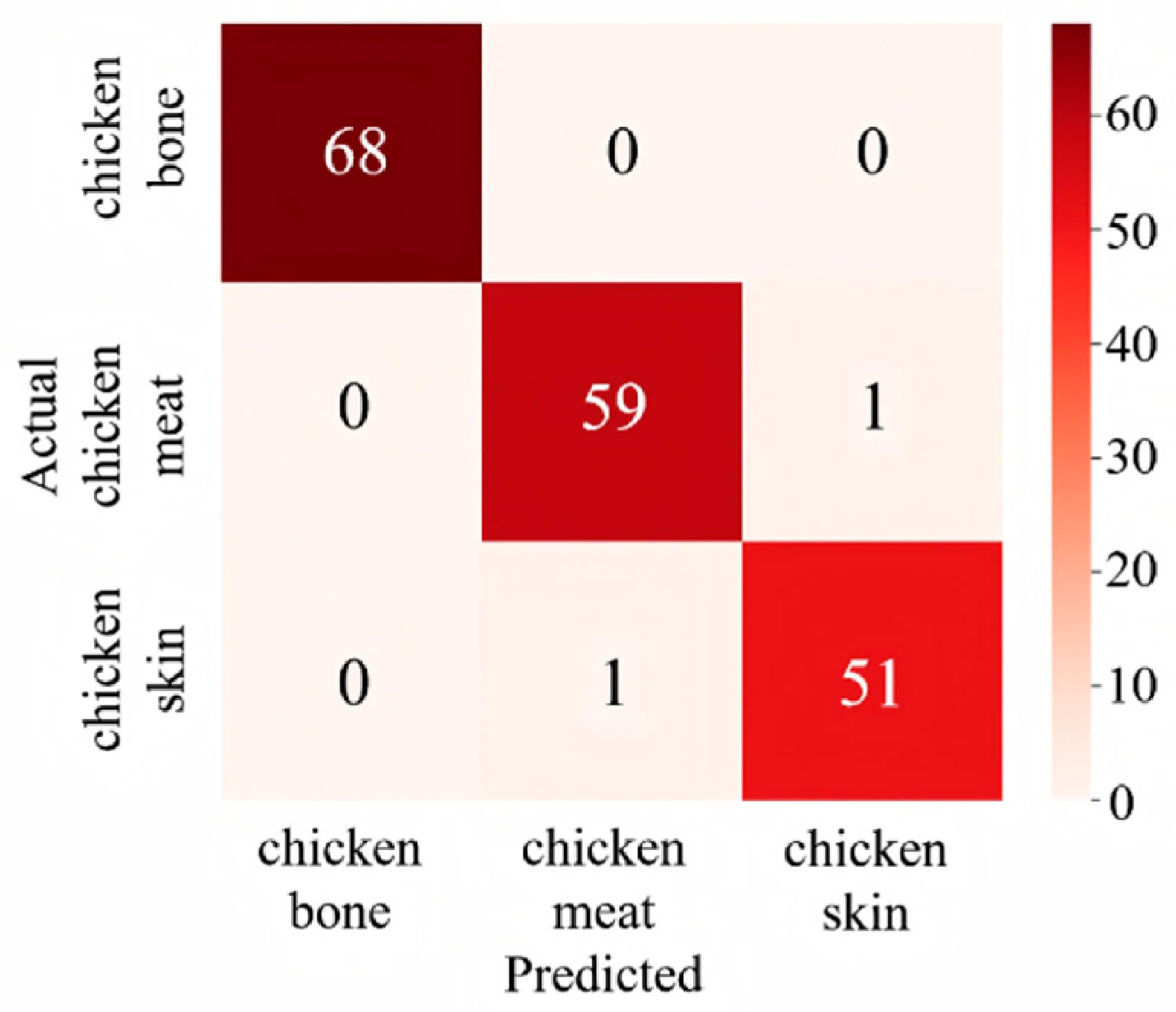
Confusion matrix of the CARS-SVM tissue classification model.

To verify the effectiveness of the CARS algorithm, this study constructed an SVM classification model using the extracted characteristic wavelengths as inputs to evaluate its performance. Figure 6 presents the confusion matrix obtained by testing the CARS-SVM model on the test set, showing that all chicken bone samples were correctly classified with zero misclassification. One chicken breast sample was misclassified as chicken skin, while one chicken skin sample was misclassified as chicken breast. This is likely due to the similarities in chemical compositions, such as protein and fat, between chicken breast and skin, resulting in closely aligned spectral signatures, which is consistent with the spectral trends shown in Figure 3. The classification results of the model are summarized in Table 1. As indicated, the SVM model based on characteristic wavelengths can accurately identify the categories of chicken breast, bone, and skin. The average precision for all categories is near 1, indicating an extremely low false-positive rate, and all recall rates exceed 0.98, suggesting a minimal risk of missed detections. Specifically, all performance metrics for the chicken bone category are 1 or near 1, demonstrating the model’s superior recognition capability for this class. These results prove that the characteristic wavelengths extracted by CARS effectively retain key information for distinguishing meat, skin, and bone while eliminating redundant bands, thereby providing a solid foundation for constructing high-quality pseudocolor images and target detection datasets. It should be noted that Table 1 shows the classification results for tissue ROI samples (meat, skin, and bone), rather than the chicken carcass defect detection dataset. Therefore, the corresponding sample sizes differ.

### 3.4. Construction of Pseudo-Color Images for Chicken Carcass Defect Detection

**Figure 7:**
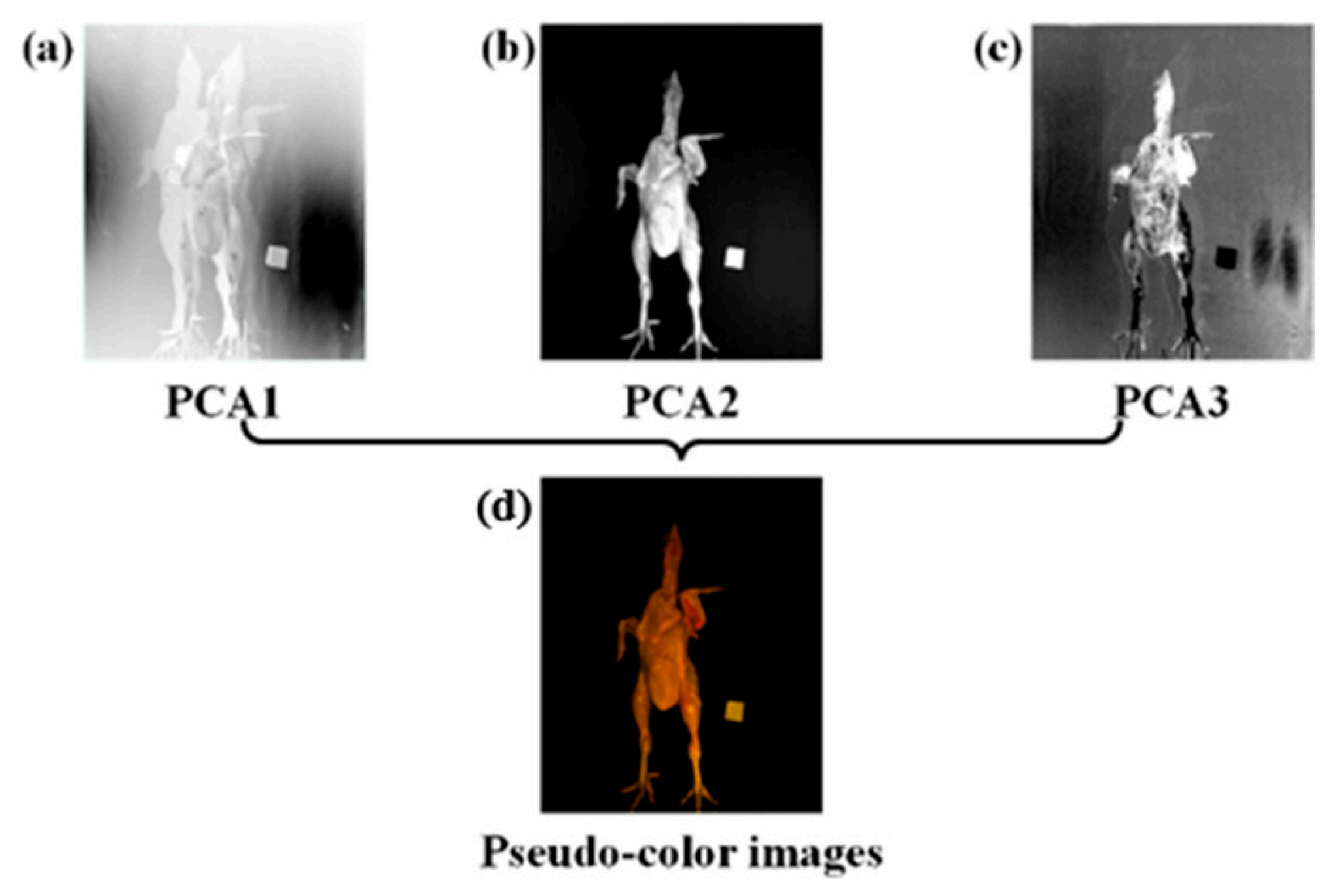
Pseudo-color Image of Chicken Carcass Defects. (**a**) First principal component image (PCA1), (**b**) second principal component image (PCA2), (**c**) third principal component image (PCA3), (**d**) false-color image.

The feature bands selected by the CARS algorithm effectively differentiate the tissue composition variations caused by different types of defects. To further enhance the visualization of different tissues in defect areas and suppress irrelevant noise, this study, based on CARS dimensionality reduction, applies PCA to process the single-channel spectral images corresponding to the 28 feature wavelengths. The main feature maps extracted by PCA are then combined to create a pseudo-color image. This pseudo-color image serves as the input for the improved chicken carcass defect detection model, ultimately achieving accurate identification and classification of skin scratches, skin break, and exposed bone on the carcass. The PCA algorithm calculates eigenvalues based on the image correlation matrix to extract the main spectral features from each image. Table 2 shows the principal component feature information of the chicken carcass hyperspectral images. The cumulative contribution rate of the first three PC images reached 96.7%, providing a balance between information retention and image dimensionality reduction, indicating that the first three PC images contain almost all the effective information of the original images, and can be used for subsequent image processing, feature extraction, and classification recognition. In this study, the first three PC images with the highest cumulative contribution rate (Figure 7a–c) were selected and combined to create a false-color image (Figure 7d). The false-color image was created by mapping the principal component images to the red (R), green (G), and blue (B) color channels. This process reduced the interference of background shadows on the chicken carcass and improved the distinction between the carcass and its background. The false-color image provided richer color contrast information, enhancing the detailed features of chicken carcass defects. This, in turn, helps improve the accuracy and robustness of the enhanced defect detection model when identifying and classifying skin scratches, epidermal peeling, and exposed bone on the carcass.

### 3.5. Improved Faster RCNN Model for Chicken Carcass Defect Detection

To enhance the recognition and classification ability of the chicken carcass defect detection model, improvements were made to the Faster R-CNN model by incorporating EPSANet50 for better feature extraction and introducing CE-FPN to improve the defect recognition performance. To validate the effectiveness of the improvements made to each module of the Faster R-CNN model in this study, ablation experiments were conducted. The experiments used the baseline Faster R-CNN model (BFR) as a reference, and the improved modules were added one by one. First, the EPSANet50 network was added to the BFR model, resulting in the FR_E50. Then, the CE-FPN was further integrated into the FR_E50 model to construct the final FR_E50_CF chicken carcass surface defect detection model. All models were trained and tested on the chicken carcass defect dataset. The performance comparison results are shown in Table 3. The FR_E50 model shows improvements in accuracy, recall, and AP compared to the BFR model, particularly with a significant increase in recall for epidermal peeling and exposed bone. This is mainly attributed to the PSA algorithm integrated into the EPSANet50 network, which can effectively capture multi-scale features from epidermal textures to bone morphology, while preserving fine details such as slight epidermal damage and bone edge exposure in deep networks. This helps the model better recognize epidermal damage and exposed bone. Due to the larger receptive field of the PSA compared to the convolutional network in the baseline model, it is better at capturing long-distance scratch features. Therefore, the improved chicken carcass defect detection model shows the largest increase in AP for skin scratches, which is also the main reason for the performance improvement in the other two defect categories. However, although the PSA algorithm improves the AP for skin scratches, its recall rate shows limited improvement. This indicates that the module focuses more on enhancing detection confidence rather than significantly improving defect detection ability. Further comparison between the FR_E50 and FR_E50_CF models shows that after introducing the CE-FPN network, the recall rate for exposed bone increased by 0.033, while the recall rates for skin lesions and skin scratches increased by 0.054 and 0.061, respectively. This indicates that the CE-FPN effectively enhanced the model’s overall recall capability. This improvement is attributed to its multi-scale feature fusion structure, which allows the model to simultaneously leverage both shallow details and deep semantic information from the image. As a result, it enhances the model’s ability to detect small-target defects such as skin break, skin scratches, and exposed bone.

In addition, based on the results in Table 3, the proposed improved chicken carcass defect detection model demonstrates excellent discriminative performance across all defect categories, with an overall MAP of 93.6%. Specifically, the exposed bone defect, with its unique spectral characteristics, achieves a detection accuracy of 92.6%, a recall rate of 96.5%, and an average precision of 94.8%, showing the best recognition performance. In contrast, chicken skin and chicken breast have higher morphological and spectral similarities (Figure 3), which results in slightly lower classification accuracy, recall rate, and average precision for skin scratches and epidermal peeling compared to exposed bones. Nevertheless, after the improvements to the model in this study, these two defect categories still achieved good recognition performance, which fully demonstrates that the enhancement of the Faster R-CNN model effectively improved its ability to detect various chicken carcass defects.

**Figure 8:**
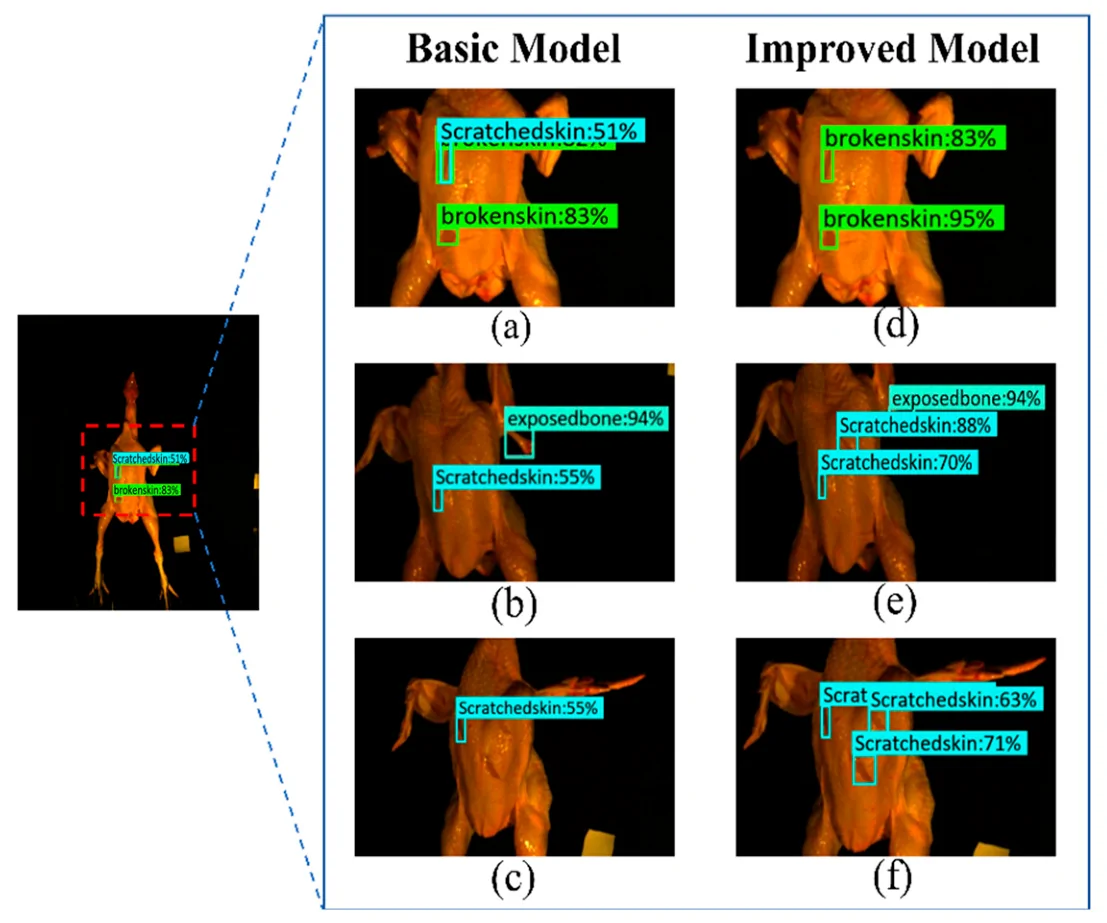
Detection results of BFR on samples of (**a**) skin break, (**b**) skin scratches, and (**c**) exposed bone, and detection results of FR_E50_CF on samples of (**d**) skin break, (**e**) skin scratches, and (**f**) exposed bone.

Figure 8 compares the detection results of the BFR model and the FR_E50_CF model for three different damage samples. By comparing Figure 8a and Figure 8d, it is evident that, compared to BFR, the FR_E50_CF model shows a significant improvement in confidence for detecting epidermal peeling and avoids false positives for skin scratches. This performance enhancement is primarily attributed to the advantage of the EPSANet50 network in extracting multi-scale features. By comparing Figure 8b and Figure 8e, it can be observed that the BFR model has a relatively high rate of false negatives for detecting skin scratches. The improved FR_E50_CF model, however, shows improvements in both recall and confidence. This enhancement is due to the introduction of the CE-FPN network, which has strengthened the model’s ability to detect small target samples. Comparing Figure 8f with Figure 8c, the improved network enhances the confidence in detecting exposed bone and also increases the detection rate of skin scratches present in the same sample. In summary, by comparing the actual performance of the models on the three types of samples, the improvements made in this study effectively address the false positives and false negatives present in the BFR model. The improved FR_E50_CF model not only maintains high precision but also enhances recall capability.

The improved Faster R-CNN model for chicken carcass defect detection proposed in this study effectively enhances the recognition and classification abilities for detecting skin scratches, skin break, and exposed bone. The enhanced feature extraction network improves the model’s perception of carcass defects, reducing both false detection and missed detection rates. Chicken carcasses from different production batches exhibit certain variations in appearance, such as skin color, which may introduce some non-target variability into hyperspectral data collection. However, the hyperspectral detection method used in this study does not rely on absolute values of surface color but focuses on capturing characteristic spectral responses caused by changes in tissue biochemical composition. Specifically, defects on chicken carcasses, such as skin tears and exposed bones, cause subcutaneous tissue, which has a different biochemical composition and is normally covered by skin, to be directly exposed to the light path. This results in spectral characteristics that exhibit more pronounced and specific differences compared to intact skin. By combining hyperspectral imaging technology with an improved Faster R-CNN model, we can effectively focus on these intrinsic spectral signals, thereby significantly mitigating the adverse effects of variations in surface color on defect detection. It should be noted that the dataset used in this study consists primarily of carcasses with defects, as the main objective of this research is to achieve precise identification and localization of defect areas, rather than binary classification between defective and defect-free carcasses. The detection process was performed on images of intact chicken carcasses, and normal regions within defective carcasses were also included in the model evaluation; thus, the model’s ability to distinguish between defect areas and intact areas can be assessed to a certain extent. Future work will focus on further optimizing feature extraction capabilities for carcass defects and integrating data processing improvements to enhance model computational efficiency, as well as refining the environmental adaptability design for online applications, laying the foundation for future industrial deployment.

## 4. Conclusions

This study proposes a chicken carcass defect detection method that integrates hyperspectral imaging and deep learning techniques, achieving effective recognition of skin scratches, skin break, and exposed bone. The method extracts spectral features from the region of interest in the hyperspectral images of chicken carcasses, and generates pseudo-color images by combining the feature bands selected through CARS dimensionality reduction and PCA fusion. This forms a feature-enhanced chicken carcass defect dataset. To improve the recognition performance of small target defects, this paper specifically modified the Faster R-CNN model. The combination of EPSANet50 and CE-FPN as the feature extraction network significantly enhances the model’s ability to capture subtle defect features and perform multi-scale fusion. The results show that, under the experimental conditions of this study, the proposed method achieved good detection performance in the task of detecting defects in chicken carcasses, with AP values for all defect categories exceeding 92%, recall rates above 91%, precision above 90%, and mAP reaching 93.6%. Compared to existing mainstream detection models, the improved Faster R-CNN chicken carcass defect detection model shows significant advantages. This validates the effectiveness of optimizing the model structure for chicken carcass defect features. This study provides an effective technical solution for automated detection of common chicken carcass defects and demonstrates the potential of hyperspectral imaging combined with deep learning for appearance quality inspection of poultry products. Nevertheless, the current study was conducted using chicken carcasses from a single breed under specific production conditions. Future studies will include carcasses with greater variability in breed, age, and feeding conditions to further evaluate the robustness and generalization capability of the proposed method in practical industrial applications.

## Figures and Tables

**Table 1:** Classification results of the CARS-SVM model for tissue ROI classification.

Model	Categories	Number of Samples Trained	Number of Samples Tested	Number of Feature Bands	AP	Recall	Accuracy
CARS-SVM	Bone	132	68	28	1	1	0.98
	Meat	140	60	28	0.98	0.98	
	Skin	148	52	28	0.98	0.98	

**Table 2:** Principal Component Feature Information of Chicken Carcass.

PC	Eigenvalue	Percent
1	3.375	68.41%
2	1.360	95.97%
3	0.035	96.70%
4	0.027	97.26%
5	0.023	97.73%

**Table 3:** Comparison of Model Performance with Different Modules Added. (“√” indicates that the corresponding network is integrated into the model).

Model	EPSANet50	CE- FPN	Defect Categories	Recall	Precision	AP	mAP
BFR			skin break	0.794	0.834	0.793	0.802
			skin scratch	0.824	0.845	0.788	
			exposed bone	0.855	0.867	0.825	
FR_E50	√		skin break	0.845	0.849	0.824	0.857
			skin scratch	0.831	0.856	0.865	
			exposed bone	0.899	0.893	0.882	
FR_E50_CF	√	√	skin break	0.937	0.903	0.935	0.936
			skin scratch	0.914	0.907	0.926	
			exposed bone	0.965	0.926	0.948	

## Data Availability

The data presented in this study are available upon request from the corresponding author. The raw data are not publicly available due to pending patent application.
